# Metabolic and Proteomic Perspectives of Augmentation of Nutritional Contents and Plant Defense in *Vigna unguiculata*

**DOI:** 10.3390/biom10020224

**Published:** 2020-02-03

**Authors:** Aqeel Ahmad, Tanveer Alam Khan, Samavia Mubeen, Iqra Shahzadi, Waheed Akram, Taiba Saeed, Zoobia Bashir, Rui Wang, Mufid Alam, Shakeel Ahmed, Du Hu, Guihua Li, Tingquan Wu

**Affiliations:** 1 Vegetable Research Institute, Guangdong Academy of Agricultural Sciences/Guangdong Key Laboratory for New Technology Research of Vegetables, Guangzhou 510640, China; aqeelahmad1@gmail.com (A.A.); tanveeralamkhan10@gmail.com (T.A.K.); meher_waheed@yahoo.com (W.A.); taibasaeed02@gmail.com (T.S.); wrui_1999@163.com (R.W.); duhu1986@163.com (D.H.); liguihua@gdaas.cn (G.L.); 2 State Key Laboratory for Biocontrol, School of Life Sciences, Sun Yat-Sen University, Guangzhou 510275, China; mubeen3@mail.sysu.edu.cn; 3 School of Resource and Environmental Science, Wuhan University, Wuhan 430072, China; iqrasardar@whu.edu.cn; 4 National Laboratory of Solid State Microstructures Department of Physics Nanjing University 22 Hankou Road, Nanjing 210093, China; zoobiabashir@gmail.com; 5 National Key Laboratory of Crop Genetic Improvement and National Center of Plant Gene Research, College of Life Science and Technology, Huazhong Agricultural University, Wuhan 430070, China; mufid.agribhu@gmail.com; 6 Instituto de Farmacia, Facultad de Ciencias, Universidad Austral de Chile, Campus Isla Teja, Valdivia 5090000, Chile; shakeel1177@yahoo.com

**Keywords:** chemical-protein docking, defense pathways, glucanase isozyme, *Macrophomina phaseolina*, nutrition metabolism, phosphoglycerate kinase 3, physicochemical analysis, plant protein modeling, protein active pockets, protein-protein interaction

## Abstract

The current study enlists metabolites of *Alstonia scholaris* with bioactivities, and the most active compound, 3-(1-methylpyrrolidin-2-yl) pyridine, was selected against *Macrophomina phaseolina*. Appraisal of the *Alstonia* metabolites identified the 3-(1-methylpyrrolidin-2-yl) pyridine as a bioactive compound which elevated vitamins and nutritional contents of *Vigna unguiculata* up to ≥18%, and other physiological parameters up to 28.9%. The bioactive compound (0.1%) upregulated key defense genes, shifted defense metabolism from salicylic acid to jasmonic acid, and induced glucanase enzymes for improved defenses. The structural studies categorized four glucanase-isozymes under beta-glycanases falling in (Trans) glycosidases with TIM beta/alpha-barrel fold. The study determined key-protein factors (Q9SAJ4) for elevated nutritional contents, along with its structural and functional mechanisms, as well as interactions with other loci. The nicotine-docked Q9SAJ4 protein showed a 200% elevated activity and interacted with AT1G79550.2, AT1G12900.1, AT1G13440.1, AT3G04120.1, and AT3G26650.1 loci to ramp up the metabolic processes. Furthermore, the study emphasizes the physiological mechanism involved in the enrichment of the nutritional contents of *V. unguiculata*. Metabolic studies concluded that increased melibiose and glucose 6-phosphate contents, accompanied by reduced trehalose (-0.9-fold), with sugar drifts to downstream pyruvate biosynthesis and acetyl Co-A metabolism mainly triggered nutritional contents. Hydrogen bonding at residues G.357, G.380, and G.381 docked nicotine with Q9SAJ4 and transformed its bilobed structure for easy exposure toward substrate molecules. The current study augments the nutritional value of edible stuff and supports agriculture-based country economies.

## 1. Introduction

*Vigna unguiculata* (L.) Walpers is an important legume crop. Due to its drought tolerance, it is a significant component of many cropping systems in dry regions and marginal areas of the tropics and subtropics [1]. The crop is one of the main sources of amino acids, vitamins, and minerals for the human population. It constitutes a natural supplement to cereal, root, and tuber staples in the African diet. The losses of insufficient yield are amplified due to accompanying reduced nutritional quality of the plant produce [2,3]. The augmentation of the nutritional profile is very important to be achieved to provide better nutrition to the consumers. Food items with more fats and less nutritional elements (junk foods) are an emerging problem in today’s society [4]. The solution is to produce agricultural crops with enriched nutritional content. Because of the less physically active lifestyle, the strategy will assist to feed enough nutrition with the least masses of food commodities. Shortly, humans will be less interested in eating bulk quantities and then have tiresome exercises to maintain their fat and body shapes [5]. Therefore, it will be more convenient to fulfill the nutritional needs of individuals by providing them nutritionally rich foods.

Besides, human efforts to feed large populations fungal pathogens are causing a dearth in food production and the deterioration of the nutrition [6]. *Macrophomina phaseolina*, a soil-borne plant pathogen, causes charcoal rot (dry root rot) disease, which limits the profitable cultivation of cowpea. A disease incidence of up to 64%, leading to complete failure of the cowpea crop, has been reported by Roberts et al. [7]. On the other side, *Alstonia scholaris* (L.) is an important medicinal and allelopathic plant, and its extracts are used to control a variety of fungal pathogens. It produces echitamine, alstonine, pleiocarpamine, O-methylmacralstonine, and macralstonine, which have been frequently reported for their antifungal properties [8,9]. Furthermore, cowpea cultivars contain some key resistance genes, e.g., PR1b, basic PR1; PR2a, acidic glucanase; PR2b, basic glucanase; Chitinase 3, acidic; Chitinase 9, basic; Osmotin-like PR5; P69A, subtilisin-like; and Metallothionein 2b-like, regulating resistance responses to *M. phaseolina* [10]. The expression of these genes is an icon of plant resistance against the invading pathogen. Further, these genes also represent their associated resistance metabolic pathways, i.e., salicylic acid (SA), jasmonic acid (JA), and phytochelatin biosynthesis (PCB) pathways. Moreover, pathogenesis-related (PR) proteins also play an important part in plant defense responses. Among these, Glucanases are essential enzymes that belong to the PR-2 family of plant proteins. These enzymes are called hydrolysis, as they cause the hydrolysis of glycosidic bonds [11]. Induction of β-1,3-glucanase in *Vigna aconitifolia* and its implications in defense responses of moth bean plants against *M. phaseolina* have been discussed by Gupta et al. [12] and Pareek et al. [13]. Defense responses of some other isozymes of glucanase have also been reported in genus *Vigna* [14,15], which indicates the probability of multiple isozymes of glucanase in the cowpea plant. The complete view of glucanase based defense responses in *V. unguiculata* has still not been revealed. Therefore, unveiling the glucanase defenses was included as one of the main tasks of the current study. It has been previously reported that the bioactive compounds alter the physiological parameters and change the nutritional contents in a plant [16]. Using the bioactive compound can pave our way toward nutritionally rich food, which would be helpful in feeding large human populations globally. In the current study, bioactive compounds from *A. Scholaris* were screened which may control *M. phaseolina* infection and improve the nutritional value of *V. unguiculata*. To understand the effect of BAC on different defense-related pathways at the genetic level, several key defense-related genes were analyzed. Moreover, to explore the regulatory mechanisms of BAC on the nutritional value of *V. unguiculata,* the most active protein (MAP) in *V. unguiculata* was isolated and functionally characterized. We generated a three-dimensional structure of MAP and identified putative residues participating in binding with other *Vigna* loci and metabolites and performed catalytic activity analysis. However, the current study was planned to screen a bioactive compound of *A. scholaris* which may control *M. phaseolina* infection and improve the nutritional value of *V. unguiculata*. During the whole study period, the steps were carried out in the following order: (i) identification of Bioactive Compound (BAC) from *Alstonia* extracts; (ii) evaluation of the effect of BAC on nutritional quality of *Vigna*; (iii) determination of the MAP in *V. unguiculata* against the BAC of *A. scholaris*; (iv) structural, physicochemical, functional, and protein–protein interaction studies of the MAP of *Vigna*; (v) analysis of the effect of BAC on key defense-related genes, defense pathways (i.e., Salicylic Acid Pathway (SA), Jasmonic Acid Pathway (JA), and Phytochelatin Biosynthesis Pathway (PCB)), and glucanase defenses in *V. unguiculata*; (vi) interaction analysis of the MAP with other *Vigna* loci and metabolites; (vii) activity analysis of the MAP after getting docked with BAC; (viii) sugar metabolism, defense metabolism, and physiological analyses of *V. unguiculata* after BAC treatment. 

## 2. Materials and Methods

Detailed information on materials and methods is provided in Supplementary Data Set 4. Defense-related genes and details of primers used for their amplification are itemized in Supplementary Data Set 4.

## 3. Results

### 3.1. Disease Incidence (%) of Charcoal Rot and Percentage Disease Control by Alstonia Extracts

The application of *A. scholaris* leaf extract in *M. phaseolina* treated *V. unguiculata* plants reduced the charcoal rot incidence in all four cultivars (Table S1). The minimum DI was recorded in cultivar Elite with ethyl acetate (7.5) and chloroform extracts (8.69), whereas maximum DI was recorded by the treatment of n-hexane (12.07) extract. Percentage disease control was improved with increasing concentration of the extracts (Table S2). The least disease control was recorded with n-hexane extracts, while the maximum percentage of disease control was observed with ethyl acetate treatment. Moreover, among sub fractionations of ethyl acetate extracts, sub-fraction FR04 was the most active sub-fraction, with the highest disease control (Supplementary Figure S1C,D). Three most active sub-fractions underwent GCMS (7000D, Agilent, CA, USA) analysis, for the identification of bioactive compound(s), as shown in Supplementary Data Set 1.

To explore the relationship between *A. scholaris* leaf extracts and percentage disease control on cultivars of *V. unguiculata*, Pearson’s correlation was adopted. The highest positive correlation (0.9418) was recorded between n-butanol extract and percentage disease control of cultivar White-Star (Table S3). The lowest correlation (0.4267) was observed between n-hexane extracts and percentage disease control of cultivar Elite. 

### 3.2. Identification of Alstonia Metabolites in Bioactive Sub-Fractions

**Figure 1:**
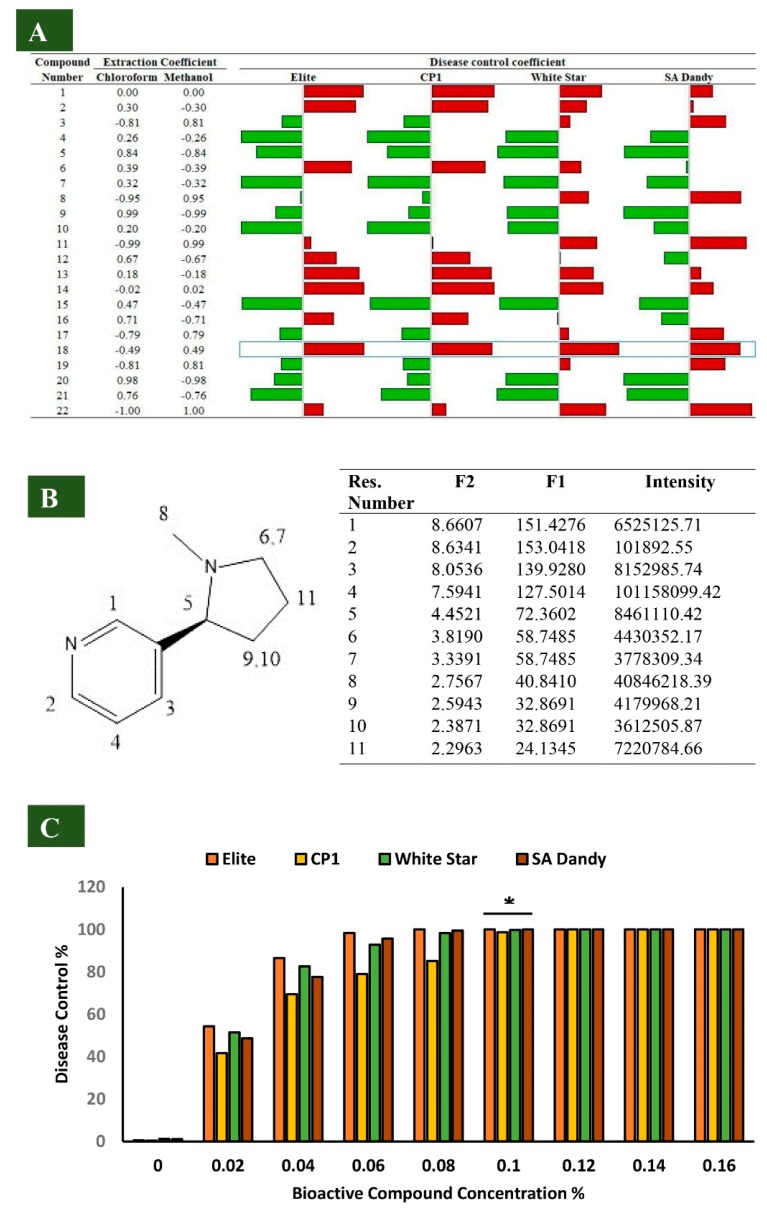
Pearson’s correlation coefficient values of each compound (01–22) with disease control are plotted in the form of horizontal bars, green bars for negative values, and red bars for positive correlation values. The extraction coefficient values are provided in the numeric form for all the compounds (**A**). Structure and value of Nuclear Magnetic Resonance (NMR) spectrometry (^1^H, ^13^C) analysis of the most active compound, 3-(1-methylpyrrolidin-2-yl) pyridine (**B**). Percentage disease control against the concentration gradient (0.00%, 0.2%, 0.4%, and 0.16%) of the most active compound, 3-(1-methylpyrrolidin-2-yl) pyridine (**C**), * *p* < 0.5.

A total of 22 compounds were detected in three most active sub-fractions of ethyl acetate (i.e., FR03, FR04, and FR05). The retention time of potentially active compounds ranged from 8 to 36 minutes. However, the bioactivity of each compound was determined from the previous literature and is mentioned in Table S4. PCC analysis identified compound nicotine, 3-(1-methylpyrrolidin-2-yl) pyridine as the most active compound against *M. phaseolina* in three sub-fractions of ethyl acetate. The extraction affinity of nicotine was 0.49 with an increasing concentration of methanol in the extraction solvent, but its affinity was −0.49 with an increasing concentration of chloroform (Figure 1A). It was easy to extract nicotine in an extraction solvent containing a high proportion of methanol (80%) and a low proportion of chloroform (20%). Moreover, values for 2D NMR ^1^H, ^13^C (Bruker, Davis, CA, USA) were analyzed and used to construct the structure of 3-(1-methylpyrrolidin-2-yl) pyridine (Figure 1B). The test cultivars were treated with different concentrations of nicotine, to determine the minimum concentration giving complete control of the disease. The assay proved 0.1% concentration of nicotine as the best concentration to be used in downstream experimentation (Figure 1C).

### 3.3. Nicotine Effect on Nutritional Contents, Physiological Factors, Enzymes, and Proline Contents

**Figure 2:**
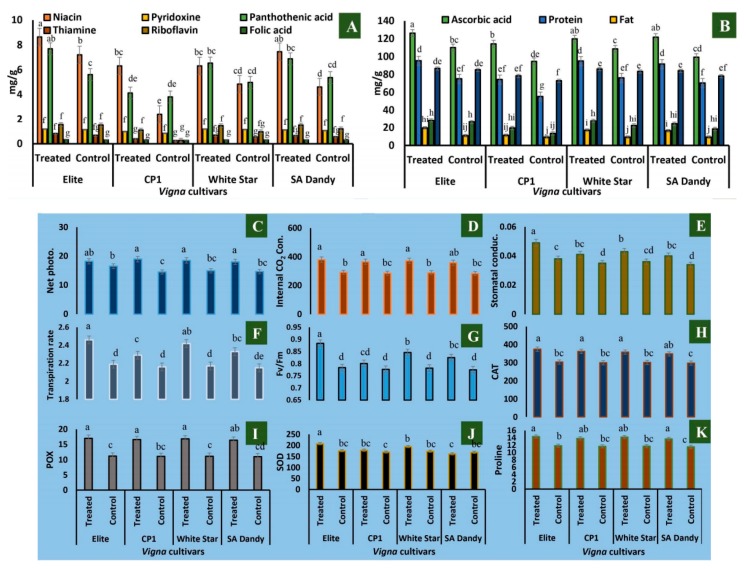
Nutritional analysis of *Vigna unguiculata* after the application of 3-(1-methylpyrrolidin-2-yl) pyridine. Values of niacin, pyridoxine, pantothenic acid, thiamine, riboflavin, and folic acid contents are provided in (**A**), while ascorbic acid, protein, fat, fiber, and carbohydrate contents are shown in (**B**). Data of nine physiological parameters are plotted as (Net photo.) net photosynthetic rate (µ mol CO_2_ m^−2^ s^−1^) (**C**), (internal CO_2_ con.) internal CO_2_ concentration (ppm) (**D**), stomatal conductance (mol H_2_O m^−2^ s^−1^) (**E**), transpiration rate (mmol H_2_O m^−2^ s^−1^) (**F**), Fv/Fm (**G**), (CAT) catalase (mmol H_2_O_2_ decomposed g^−1^ FM) (**H**), (POX) peroxidase (units g^−1^ (FM)) (**I**), (SOD) superoxide dismutase (units g^−1^ (FM)) (**J**), and proline (µ mol g^−1^(FM) (**K**). The experiment was replicated thrice, and mean values of nutritional contents were calculated to construct the plots.

Elite exhibited the maximum quantities (mg/g) of all the nutritional factors (niacin, 7.2; pyridoxine 1.13; pantothenic acid, 5.61; thiamine, 0.67; riboflavin, 1.52; folic acid, 0.31; ascorbic acid, 110.21; protein, 75.19; fat, 10.92; fiber, 26.75; and carbohydrates, 85.24 mg/g). Elite contained the highest nutrition contents; however, SA Dandy bared the least. The application of nicotine caused 5%–18% induction of nutritional contents in cultivar Elite, and a comparable nutrition elevation was observed in cultivar White Star (Figure 2).

Net photosynthetic rate (*P_N_*) and its attributes (internal CO_2_ concentration, stomatal conductance, transpiration rate, and Fv/Fm), antioxidant enzymes (CAT, POX, and SOD), and proline contents were significantly increased when treated with nicotine in all four *V. unguiculata* cultivars, as compared to their respective control (Figure 2C–J). The maximum increase in all physiological parameters was noticed in Elite cultivar, resulting in 10.36% (*P_N_*), 28.94% (*g_s_*), 30.58% (*C_i_*), 22.83% (*E*), and 25% (Fv/Fm) increase over their respective control (Figure 2C–G). 

Likewise, the maximum enhanced activities of CAT (23.42%), POX (51.69%), and SOD (17.61%) were found in Elite cultivar, as compared to their respective control plants. 

The maximum accumulation of proline (21.31%) was found in the Elite cultivar treated with nicotine as compared to control plants.

### 3.4. Modeling of the Most Active Protein and Its Interactions

**Figure 3:**
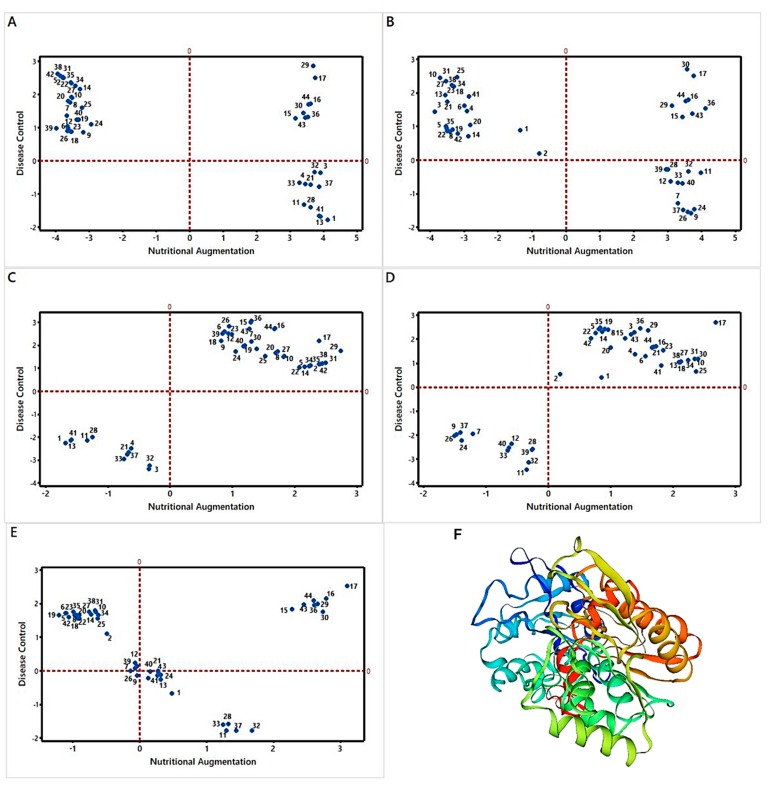
Screening of the most active protein (MAP) of *Vigna unguiculata* through principal component analysis (PCA) against the treatment of bioactive compound 3-(1-methylpyrrolidin-2-yl) pyridine. The X-axis coefficient represents the affinity of individual proteins with augmentation of nutritional contents; however, the coefficient value on Y-axis is the interrelation of proteins with disease control. A total of 44 differentially expressed proteins are mentioned in the matrix plot of cultivar Elite (**A**), CP1 (**B**), White Star (**C**)**,** and SA-Dandy (**D**). The average behavior of protein species with reference to bioactivities in all the four *Vigna* cultivars (**E**). The bilobed structure of screened MAP phosphoglycerate kinase 3-Q9SAJ4 (**F**).

The protein profiles of the tested *V. unguiculata* cultivars highlighted a total of 44 proteins in response to nicotine applications. All the proteins belonging to different metabolic functions were performing diverse physiological activities according to the data consulted from the protein database UniProt (Table S5). Principal component analysis of proteins revealed Phosphoglycerate kinase 3 (PGK3) as one of the most active proteins against nicotine treatments, hereby causing nutritional augmentation and disease control. The homology modeling of PGK3 structure was performed by online software I-TASSER (https://zhanglab.ccmb.med.umich.edu/). The predicted model of PGK3 was monomeric in nature. The structure was distinctly bilobed with a transited depressed region between the two lobes. The domains were clearly connected at two locations: (i) beta beta-sheet L, residues 191-202 and (ii) alpha alpha-helix 13. The structure possessed an open-to-close transition in a hinge bending manner (Figure 3F).

**Figure 4:**
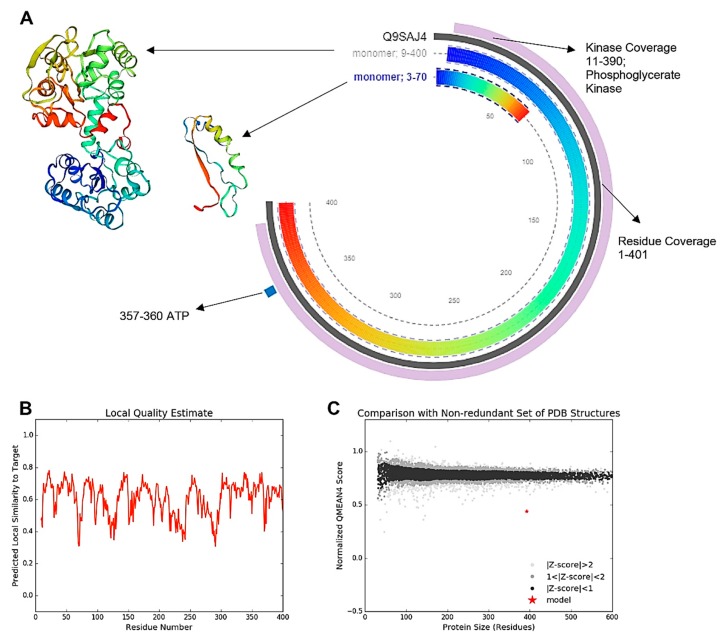
Tertiary and primary structure of the most active protein species, Q9SAJ4, showing its two constituting monomers ranging 9-400 residues for monomer 1, and 3-70 residues for monomer 2 (**A**). Similarity chart for protein Q9SAJ4 structure with the target protein model (**B**). Energy-distribution plot QMEAN4 score comparing with a non-redundant set calculated at Z-Score >2 (**C**).

The ATP associated site consisted of four residues, 357-360, and kinase activity was detected between 11 and 390 residues. The local similarity to target protein model ranged from 0.3 to 0.8, and QMEAN4 score fell >0.7 while comparing with a non-redundant set calculated at Z-Score >2 (Figure 4).

**Figure 5:**
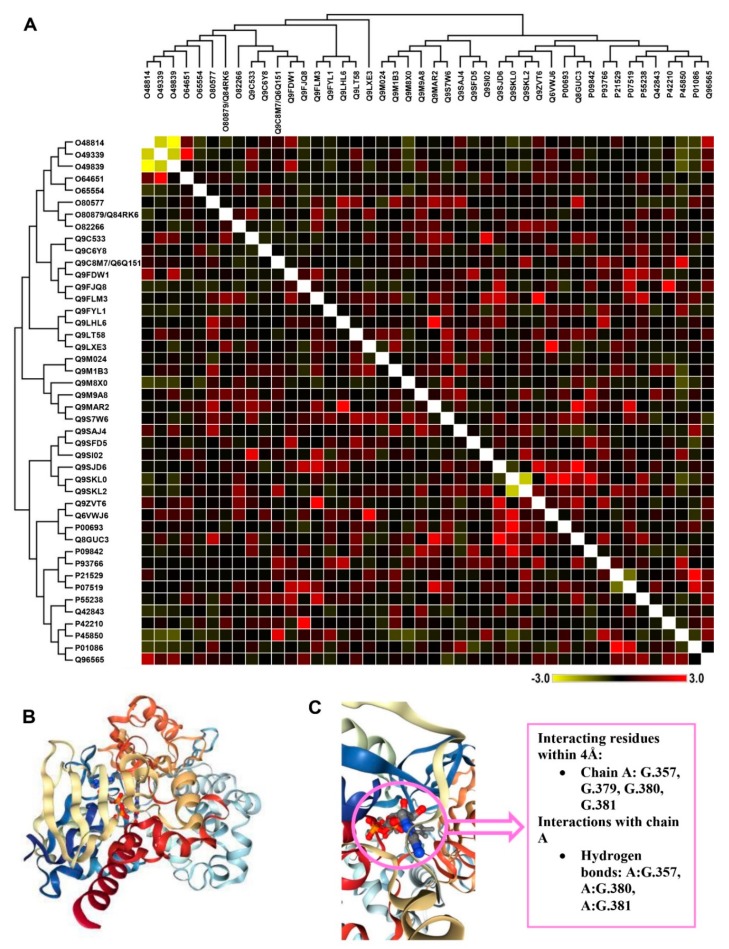
The co-expression interaction of all the differentially expressed protein species in *Vigna unguiculata* (**A**). The evolutionary tree on the left side was constructed by using the Neighbor-Joining method. The optimal tree had the sum of branch length = 51.83488287, whereas the evolutionary distances were computed by using the Poisson correction method and were in the units of the number of amino acid substitutions per site. All ambiguous positions were removed for each sequence pair (pairwise deletion option). The dendrogram on the upper side was plotted by using the Maximum Likelihood method and the JTT matrix-based model. The tree had the highest log likelihood (-56253.26). The tree for the heuristic search was obtained automatically by applying Neighbor-Join and BioNJ algorithms to a matrix of pairwise distances, estimated by using a JTT model and then selecting the topology with superior log likelihood value. Evolutionary analyses were conducted in MEGA X. The chemical protein docking was drawn between Q9SAJ4 and 3-(1-methylpyrrolidin-2-yl) pyridine (**B**). The close-up of the chemical protein docking with the details of interacting residues (**C**).

In protein-the co-expression interaction of protein interactions, it was found that PGK3 inversely interacts with SNF1-related protein kinase (SnRK), an AKIN gamma protein. However, a positive interaction was found between protein PGK3 (Q9SAJ4) and Oxalate oxidase 1 (P45850), which is a hydrogen peroxide releasing protein in the apoplast. A similar direct interaction of DNA binding transcription factor was observed with PGK3 (Figure 5A). The size of interacting residues within protein PGK3 is 4Å, all situated on chain A (G.357, G.379, G.380, and G.381). The hydrogen bonding profile of chain A enabled it to interact with nicotine at residues G.357, G.380, and G.381 (Figure 5B,C).

### 3.5. Expression of Defense-Related Genes in V. unguiculata

**Figure 6:**
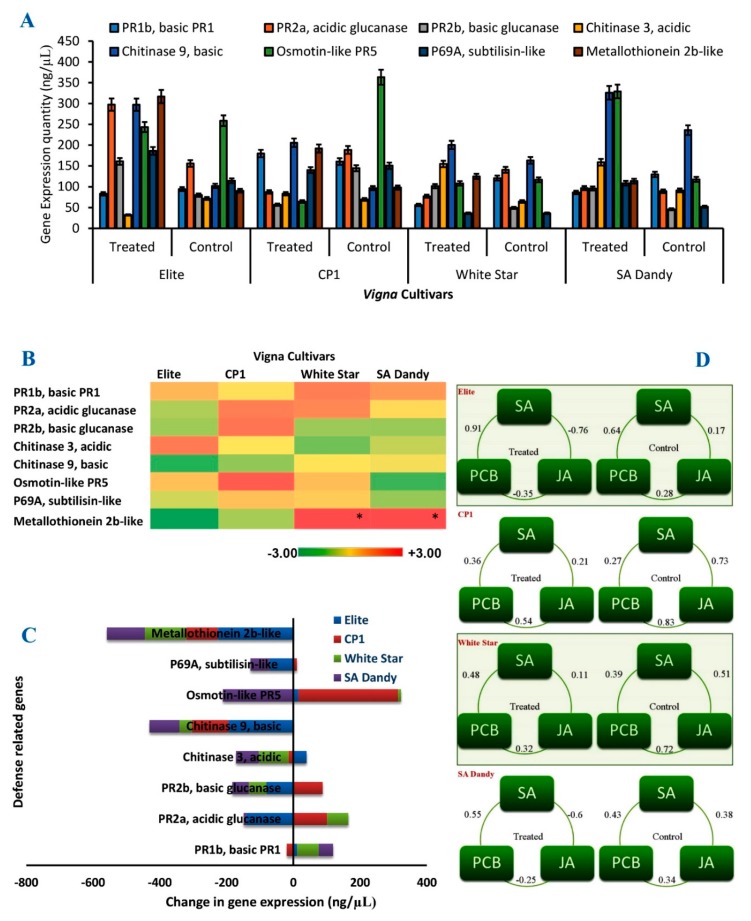
Expression analysis of defense genes in four *Vigna* cultivars (**A**). Heatmap showing the extent of upregulation or downregulation of defense genes after the application of bioactive compound (**B**), *p* < 0.5. Composite change in the expression of defense-related genes in *Vigna* plants after the application of bioactive compound (**C**). Interrelation among the defense-related metabolic pathways (salicylic acid, SA; jasmonic acid, JA; and phytochelatin biosynthesis, PCB) before and after the treatment of the bioactive compound (**D**).

The most abundantly transcribed defense genes in *Vigna* were Chitinase 9 basic, Osmotin-like PR5, and Metallothionein 2b-like. The maximum expression of Metallothionein 2b-like was found in cultivar Elite, while Osmotin-like PR5 was the maximum transcribed gene in cultivar CP1 (Figure 6A). However, Cultivars Elite, White Star, and SA-Dandy showed upregulation in gene expressions with nicotine treatment, as compared to control. These three cultivars also showed upregulation of the Osmotin-like PR5 gene, whereas the expression of Metallothionein 2b-like gene was downregulated in all the four cultivars after the application of ethyl acetate extracts (Figure 6B,C). Moreover, in White Star and SA-Dandy cultivars, PR2b (basic glucanase) and Chitinase 3 acidic genes were upregulated with ethyl acetate extracts application, whilst treatment initiated transcription of Metallothionein 2b-like gene, which was not being transcribed in control plants.

### 3.6. Correlation between A. scholaris Extracts and Defense-Related Pathways

Pearson’s correlation analysis was adopted to explore the relationship between *A. scholaris* extracts and defense-related pathways, such as salicylic acid (SA) pathway, jasmonic acid (JA) pathway, and phytochelatin biosynthesis pathway (PCB). When nicotine treatment was given to the Elite plant, the correlations between SA and JA pathways (PCC = −0.76) and between JA and PCB pathways (PCC = −0.35) were negative. However, the correlation between PCB and SA pathways were positive and strong (PCC = 0.91 in treatment and 0.64 in control).

In control treatments, all of them were slightly positively correlated (PCC = 0.17 and 0.28). In nicotine-treated cultivars CP1 and White Star, the correlation between SA, JA, and PCB pathways were positive in tested (PCC = 0. 21, 0.54, 0.36 and 0. 11, 0.32, 0.48) and control (PCC = 0.73, 0.83, 0.27 and 0.51, 0.72, 0.39) treatments, respectively, whereas the correlation is stronger under control treatment. Moreover, when cultivar SA-Dandy treated with nicotine, the correlations between SA and JA and between JA and PCB pathways were negative (PCC = −0. 60, −0.25), and between PCB and SA, the correlation was positive (PCC = 0.55) (Figure 6D). 

### 3.7. Glucanase Isozymes and Their Physiochemical Analysis

**Figure 7:**
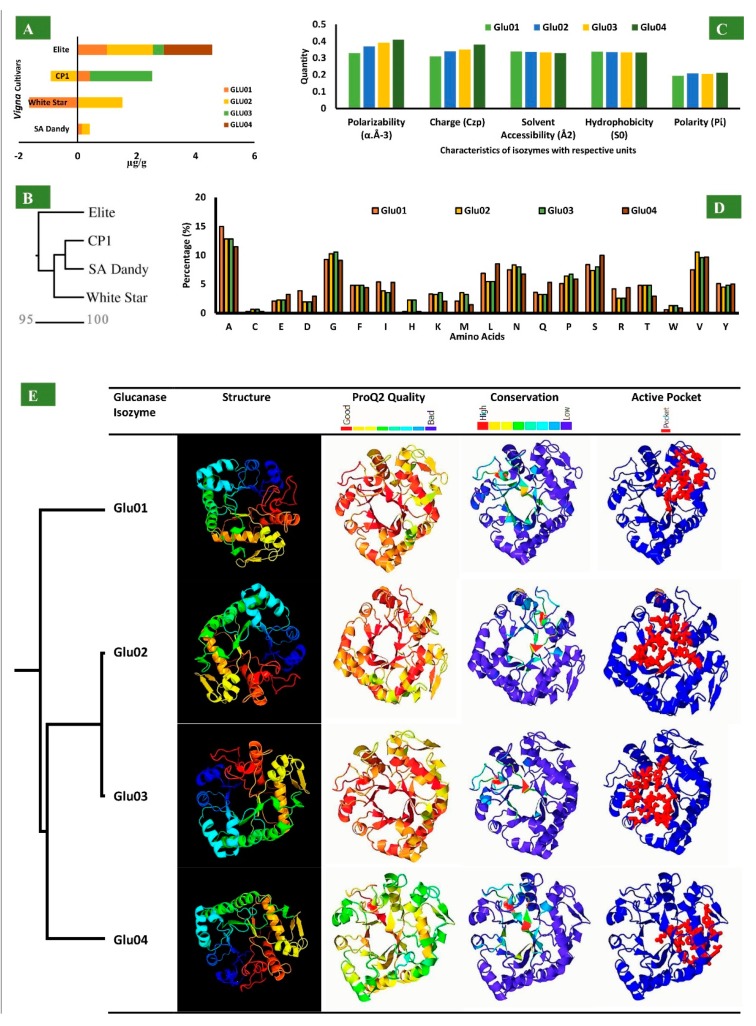
Alteration in the expression of glucanase isozymes in each cultivar of *V. unguiculata* after the application of 3-(1-methylpyrrolidin-2-yl) pyridine (**A**). Dendrogram of four cultivars of *Vigna unguiculata* based on the differences in glucanases (**B**). Physiochemical characteristics of the glucanase isozymes (**C**). The percentage share of each amino acid in the composition of the glucanase isozymes (**D**). Three-dimensional models of glucanase isozymes (Glu01, Glu02, Gl03, and Gl04) homologated with PSI-Blast (**E**). Secondary structure and disorder were calculated with Psi-pred and Diso-pred for the construction of a hidden Markov model. The largest pocket detected is shown in wireframe mode, colored red. The ProQ2 quality assessment algorithm was used to determine the quality of protein structure. The evolutionary history was inferred by using the UPGMA method. The optimal tree was drawn to scale, with the same branch lengths as those of the evolutionary distances used to infer the phylogenetic tree. The evolutionary distances were computed by using the Poisson correction method. All ambiguous positions were removed by the pairwise deletion method.

A total of four isozymes of glucanase (Glu01, Glu02, Glu03, and Glu04) were detected in *V. unguiculata* cultivars, and Glu01 was detected only in cultivar Elite. Glu02 and Glu03 were detected in treated and control plants of all four cultivars. Glu04 was detected only in treated plants of cultivars Elite and CP1. The expression of Glu03 was usually lower than other glucanases. All the four glucanases were upregulated in cultivar Elite, after the application of BAC. However, a similar treatment could induce two glucanases, Glu01 and Glu03, in cultivar CP1, and Glu01 and Glu02 in SA-Dandy. Cultivar White Star showed an elevation in only one glucanase isozyme (Glu02) after being exposed to 3-(1-methylpyrrolidin-2-yl) pyridine. Whereas, the same treatment downregulated one glucanase in each of the two cultivars CP1 and White Star (Figure 7A). The dendrogram based on the expressions related data of glucanases showed CP1 and SA-Dandy as the most closely related cultivars, with almost 98% similarity, which placed them in a single group. Elite cultivar had a 95% similarity to other studied cultivars (Figure 7B).

In this study, different physiochemical analyses of the four isolated glucanases were performed. Glu04 exhibited the maximum polarizability (0.41), charge (0.38), and polarity (0.21). However, in terms of hydrophobicity and solvent accessibility, Glu01 had the maximum values, and Glu04 possessed the least values (Figure 7C). The composition of the amino acid sequences of all the four glucanases was different from each other. Alanine was the most abundant amino acid in all the isozymes. Alanine residues were 14.97%, 12.82%, 12.82%, and 11.47% of the total residues of Glu01, Glu02, Glu03, and Glu04, respectively. Furthermore, cysteine contributed the least share in building glucanase isozymes, with 0.299%, 0.64%, 0.64%, and 0.294% residues in Glu01, Glu02, Glu03, and Glu04, respectively (Figure 7D).

The three-dimensional structure of all four glucanases was determined by molecular modeling, using online prediction software I-TASSER version 5.1 (Ann Arbor, Michigan, USA). The predicted protein model of Glu01 showed homology with TIM beta/alpha-barrel fold, which falls in the superfamily: (Trans) glycosidases, and family: beta-glycanases. However, the Glu02 model based on the single highest scoring template contained 306 residues, providing 98% sequence coverage. The homology model of Glu03 consisted upon residues number of 312 was displayed in the category of beta-glycanases classified under (Trans) glycosidases. The constructed model was 98% homologous to a template TIM beta/alpha-barrel. The homology model of Glu04 was a beta-1,3-glucanase, with a hydrolase header, and consisted of a total of 340 amino acid residues. Collectively, 310 residues constructed a homologous model with 100% confidence, placing the Glu02 and Glu03 at the closest place in the phylogenetic dendrogram of isozymes (Figure 7E).

### 3.8. Physiological Interactions

**Figure 8:**
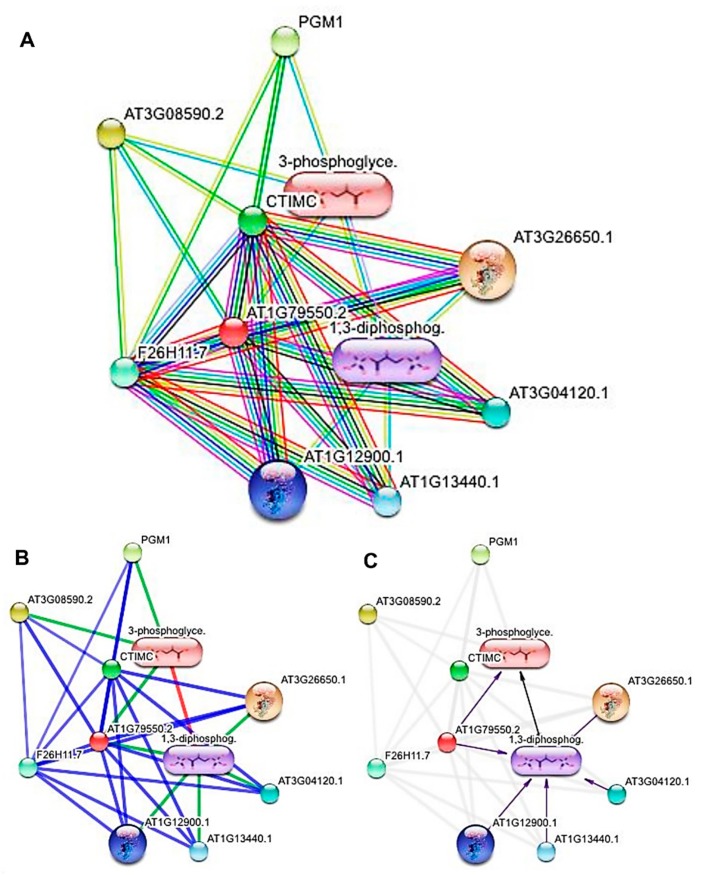
The evidence view showing the binding affinities of Q9SAJ4 domain with other biochemicals (**A**). Different line colors represent the types of evidence for the association, as shown in Table S6; Supplementary Data Set 1. The confidence view representing the strength of ligand associations (protein–protein, chemical–protein, and chemical–chemical interactions), using different weight lines (**B**). Stronger associations are shown by thicker lines. Protein–protein interactions are shown in blue, chemical–protein interactions in green, and interactions between chemicals in red. Actions view depicting the modes of action in different colors (**C**).

PGK3 directs reverse pathways of glycolysis, and in gluconeogenesis and optimized biochemical conditions, the glycolytic direction of the pathways is favored. In plants, PGK3 catalyzed the phosphorylation process of 1,3-bisphosphoglycerate (1,3 BPG) and ADP, which produced phosphoglycerate 3 (PG3) in the Calvin cycle, whereas ribulose-1,5-bisphosphate (RuBP) was generated as a part of the reaction (Supplementary Figure S4). However, the evidence view displayed the binding affinities of PGK3 conserved domain with other biochemicals forming a network of multiple nonsteroidal anti-inflammatory drugs (NSAIDs). The confidence view showed the strength of ligand associations (protein–protein, chemical–protein, and chemical–chemical interactions), using lines of different weights (Figure 8). The compound 1,3 BPG possessed relatively higher binding affinities than 3-phosphoglyceric acid (3PG) (Figure 8A). Moreover, 3PG was found associated with three proteins, i.e., AT3G085590.2, PGM1, and AT1G79550.2, whereas 1,3 BPG was to be found linked with AT1G79550.2, AT1G12900.1, AT1G13440.1, AT3G04120.1, and AT3G26650.1. Both the chemicals (3PG and 1,3 BPG) were also found under the mutual influence of each other (Figure 8B). Furthermore, the actions view showed the modes of action in different colors. Only the PGK3 (AT1G79550.2) was observed inducing 3PG. However, 1,3 BPG was found to be induced by five loci, i.e., AT1G79550.2, AT1G12900.1, AT1G13440.1, AT3G04120.1, and AT3G26650.1. Moreover, 3PG was also elevated by 1,3 BPG contents (Figure 8C).

### 3.9. Activity of PGK3

**Figure 9:**
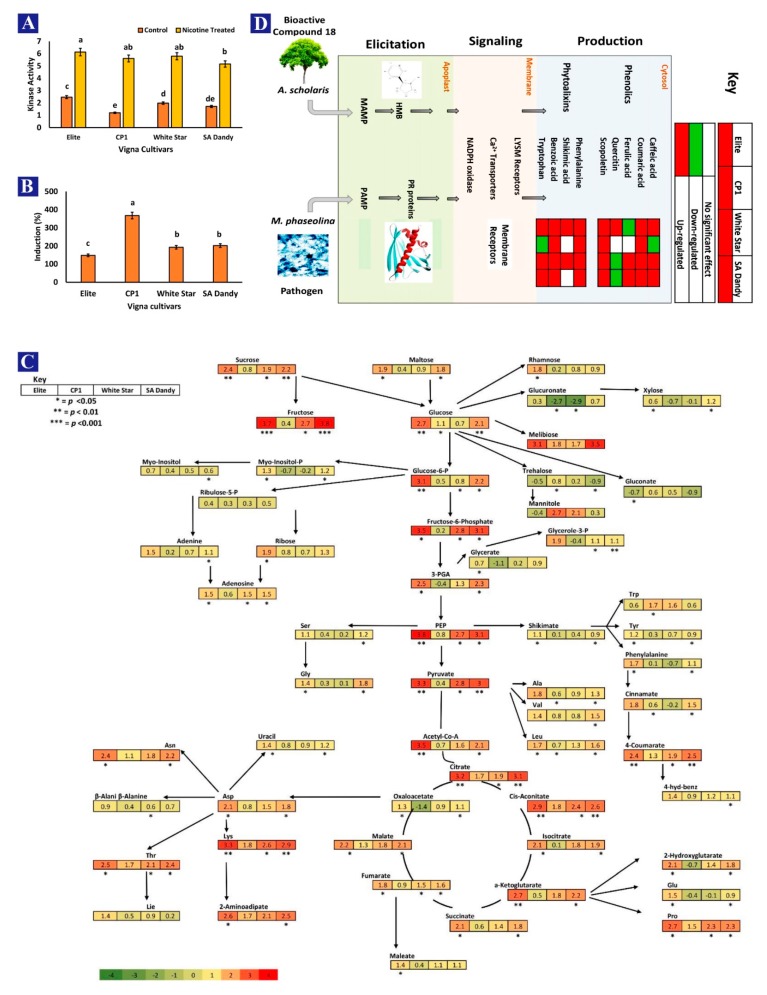
Quantification of the kinase activity of phosphoglycerate kinase 3 (PGK3) in four cultivars of *Vigna unguiculata*, after and before the application of bioactive compound nicotine, 3-(1-methylpyrrolidin-2-yl) pyridine (**A**). Percentage increase in the kinase activity of PGK3 due to the application of nicotine on four *Vigna* cultivars (**B**). Sugar metabolism of *V. unguiculata* cultivars after the application of 3-(1-methylpyrrolidin-2-yl) pyridine (**C**). Signaling of plant defense responses and *Vigna* plant defense factors after the treatment with 3-(1-methylpyrrolidin-2-yl) pyridine (**D**).

Amongst the control plants, the activity of PGK3 was recorded maximum (2.47) in cultivar Elite, followed by White Star (1.98), which was further enhanced by the treatment of nicotine (Figure 9A). The maximum induction of kinase protein (367.5%) was recorded in CP1 cultivar (Figure 9B). 

Sugar contents in the metabolism of cultivar Elite and SA-Dandy were more abundant than cultivar CP1 and White Star. In the sugar metabolism pathway, more melibiose and glucose 6-phosphate (G6P) contents were upregulated in cultivar Elite and SA-Dandy than in cultivar CP1 and White Star. A significantly higher glucose drift was observed in cultivar CP1 and White Star toward sugar species with less involvement of defense pathways, e.g., rhamnose (0.8) and trehalose (0.2). However, the cultivars with better constitutive defenses, Elite and SA-Dandy, showed −0.5 and −0.9 trehalose contents. At the stage of pyruvate biosynthesis and acetyl Co-A metabolism, only cultivar Elite showed the efficient drift of sugar species to downstream steps. The same cultivars, Elite and SA-Dandy, downregulated the sugar metabolism in the steps following the shikimate pathway. The quantities of amino acids originating from the α-ketoglutarate side chain were also abundant (Figure 9C). The complete annotations are provided in Supplementary Data Set 3. Moreover, increased phytoalexin quantities were recorded in the *Vigna* cultivar Elite during the post-treatment period of BAC. The contents of only one phenolic compound (ferulic acid) were found to be downregulated in Elite. However, quercetin contents showed a significant decrease in cultivar White Star. Collectively, two defense factors (tryptophan and caffeic acid) were downregulated in cultivar CP1 (Figure 9D).

**Figure 10:**
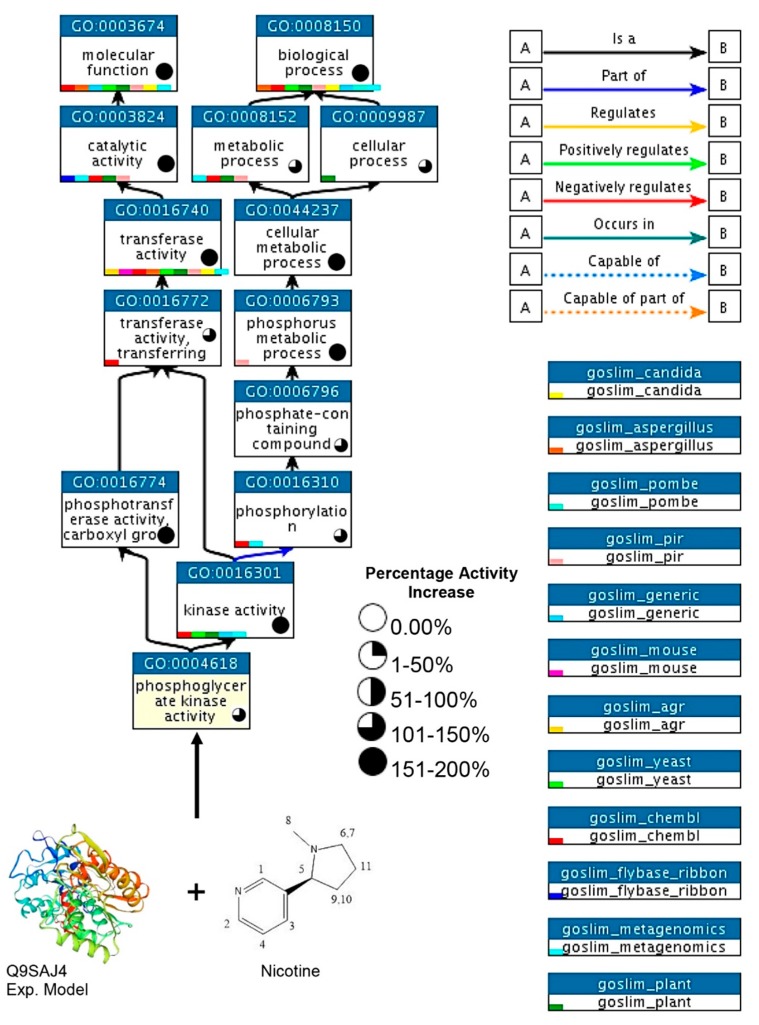
Role of phosphoglycerate kinase activity of Q9SAJ4 in the form of a chain of connective events and the elevation of kinase activity after the application of nicotine treatment.

In a chain of connected events, PGK3 was responsible for elevated kinase activity, as well as the phosphotransferase activity of carboxyl groups. However, when adopting the same process, it was also involved in the regulation of phosphorus metabolic processes and the transferase activity side by side in a cell. Moreover, accompanying nicotine molecules phosphotransferase activity was promoted up to 200%; however, the phosphorylation was elevated between 101% and 150%. The transferase activity further promoted catalytic processes up to 200%, which caused >150% elevated molecular functions of the cell. Furthermore, the phosphorus metabolic process was the key factor which caused an increase of up to 150% in all the metabolic and cellular processes of the cell (Figure 10). 

## 4. Discussion

The findings of the current investigation proved *A. scholaris* as a natural source of compounds with diverse bioactivities, including the control of *M. phaseolina*. The detected multiple bioactive compounds highlighted *Alstonia* extracts as a precious bioactive cocktail [17]. However, ethyl acetate was the most efficient organic solvent for the extraction of bioactive compounds from *A. scholaris* leaves [18]. The advantage of ethyl acetate lies in its easy handling at room temperature, without any special arrangements and extra costs [19]. Conversely, the SA pathway was proved to be a constitutive plant defense mechanism that protected *V. unguiculata* from *M. phaseolina* in situ. Moreover, the induction of the JA pathway to control *M. phaseolina* was observed, and dominance of the JA pathway suggests limitations of the SA pathway and successful contribution of the former in defense response. Recently, Khan et al. [20] also reported a significant contribution of the JA pathway in *Vigna* against *M. phaseolina*. Furthermore, in a parallel study, Sharma et al. [21] revealed that the tolerant cultivars exhibited a more active JA pathway than the susceptible cultivars. Therefore, the induction of the JA pathway by applying 3-(1-methylpyrrolidin-2-yl) pyridine from *Alstonia* can be proved as a practical and useful technique in the future.

The increased gas exchange elements and antioxidant enzymes are the signs of more smooth and stable plant metabolism. It indicates the safe use of nicotine as a resistance inducer without adversely affecting nutrition and vitamin contents. External applications of nicotine lead toward a complicated metabolism drift, resulting in an entirely new balance among pathways. The study has, for the first time, reported the improved nutritional value of food commodities after the treatment of a BAC. However, the net photosynthetic rate (*P_N_*) and its related attributes, i.e., internal CO_2_ concentration, stomatal conductance, transpiration rate, and Fv/Fm, were significantly increased when treated with nicotine in all four *V. unguiculata* cultivars, as compared to their respective control (Figure 2C–G). The possible reason behind the nicotine-mediated increase of photosynthetic efficiency is the elevated carboxylation rate of Rubisco and initial Rubisco activity, but it had no effects on the total activity of Rubisco. It indicates that nicotine mainly regulates the activation state of Rubisco, possibly through the action of Rubisco activase. Furthermore, to cope with various unfavorable conditions, plants have a well-developed network of the naturally evolved antioxidant systems to scavenge overproduction of reactive oxygen species. Results of the present study indicate that the activity of various antioxidant enzymes like CAT, POX, and SOD increased significantly in plants treated with nicotine (Figure 2H–J). Nicotine may induce transcripts-encoded proteins with functions such as metabolism, energy, protein destination and transport, cellular organization and biogenesis, cell rescue of defense, and transcription. Furthermore, proline acts as an osmoprotectant membrane stabilizer, and nicotine mediates the accumulation of proline, resulting in increased tolerance of plants.

Two genes, i.e., *PR2a*, acidic glucanase, and *Metallothionein 2b-like*, were found to be the primary genes involved in plant resistance, according to the results of the current study. The cultivars with higher expression of *PR2a*, acidic glucanase gene, and *Chitinase 3* gene also faced the least DI (Figure 6). Further, the study explored the induction of isozymes of glucanase, e.g., Glu04, which has not been previously reported. The results of the glucanase isozyme assay were also in accordance with the gene expression profile. *Vigna* plants with more intense isozyme bands also possessed the higher expression of *PR2a*, acidic glucanase gene, and the least DI. Moreover, the mechanism of the enhanced transcriptional level of these genes still needs some separate studies. By understanding the mechanisms of enhanced expression of *PR2a*, acidic glucanase, we could be able to develop new cowpea cultivars with improved antifungal resistance [22]. In the dendrogram (Figure 7B), cultivars CP1 and SA-Dandy were plotted in a closer phylogenetic relation than their relation with cultivar White Star. It all argues that Elite has a different enzymatic profile responsible for its least DI in comparison to the cultivars. Due to its least DI, cultivar Elite was the best choice to be cultivated in *Macrophomina* infested areas, while CP1 and SA-Dandy must be the least priority. All these cultivars varied in the expression of glucanase defenses. The higher expression of glucanases might be the possible reason for the strengthened antifungal potential of *Alstonia* extracts in the case of cultivar Elite.

In the bilobed structure of PGK3, the active site constitutes two monomeric domains. The N-terminal domain makes a basic region suitable for the binding of 3PG and 1,3-BPG, while the C-terminal domain provides compatible support to the nucleotide substrates, ATP and ADP. The swinging together the formation of the two lobes of protein has been reported upon binding of any substrate molecules at the active sites, due to change in protein’s conformation [23]. The suitable hydrophobic, water-free chamber is developed after swinging apart the conformation of the protein, which prevents ATP hydrolysis [24]. A salt bridge between Arg 62 and Asp 200 uses the beta-sheet L for a hinge of this conformational change [25]. The current study has detected multiple hydrogen-bond interactions between the two domains, facilitating binding of the substrates on both domains, which clues the conformation hinge movement of the bilobed structure. Furthermore, the details of two monomeric domains have been disclosed, along with their residual lengths, kinase coverage, and site for ATP binding.

Lallemand et al. [26] showed a mimicry of phosphate ion during open-to-close confirmation of phosphoglycerate kinase. However, the connection site supporting closed conformation was reported at alpha-helix 14 and 15 ranged from residues 404 to 408. The enzyme reported in the current investigation has a residual coverage of 401; hence, the structural difference has displaced the second connection at alpha-helix 13. The open conformation was upon the release of phosphoglycerate and ATP, whereas the closed conformation contained phosphoglycerate and ADP [24]. Moreover, the reduced connection resulted in an increased area between the two lobes and easier exposure of the enzyme toward the substrate, which could be the possible reason for increased kinase activity following nicotine treatment. Furthermore, phosphoglycerate kinase consists of four hydrogen bonds and six salt bridges between two C-terminal domains [27]. Zheng et al. [27] modeled small molecules and concluded an unstable interface between phosphoglycerate kinase and the ligand molecule. However, the ligand molecules played an important role in defining structural conformation, but no biological relevance of conformational rearrangement was found in that study. The current investigation adds a functional relevance of conformation alterations in the form of enhancing kinase activity, resulting in augmented nutritional contents of *Vigna*. However, the glucanase isozymes detected in this study belong to the β-1,3-glucanase family, which is comprised of 50 reported genes in *Arabidopsis thaliana*. Forty-four genes form this large family has been grouped into 13 expression clusters (denoted A-M). The first four groups of them (A–D) contain glucanase isozymes localized to leaves [28]. The glucanases in the current investigation have been observed in the leaf tissues of *Vigna*. Therefore, they can be assumed as belonging to the first three groups (A–D). The upregulated translation of these isozymes is a significant feature observed after fungal attacks [29]. Similar behavior was also observed regarding Glu01, Glu02, Glu03, and Glu04 after the attack of *M. phaseolina*. The bioactive compound 3-(1-methylpyrrolidin-2-yl) pyridine belongs to the organic compounds kingdom and falls in class pyridines, which are stable compounds with fast penetration due to aromatic heteromonocyclic properties. A diluted dose (0.1%) of the bioactive compound was responsible for the induced expression of glucanase isozymes to hinder the attack of *M. phaseolina.* It proved the study of Vidhyasekaran [30] in which micromolar doses showed antifungal activity against a broad range of plant pathogens. Moreover, the induction of glucanases in combination with other antifungal genes and defenses is a plausible strategy to develop durable resistance in crop plants against fungal pathogens [11]. Furthermore, when compared to other defense-related enzymes, the β-1,3-glucanase activation in *Vigna* plants was the fastest and the most intensive. In *V. unguiculata*, the β-1,3-glucanase is the most active defense-related enzyme which gets activated at the imbibition stage of quiescent dry seeds germination [14,15]. However, the present study emphasizes the enhanced functionality of the glucanases by improving their spectral range and quantity of individual glucanases. 

Furthermore, glucose breakdown and synthesis are among the essential processes in every living organism and provide the required substrates for aerobic and anaerobic metabolism. In plants, the metabolism of glucose is primarily controlled by PGK3, which runs the glycolysis and gluconeogenesis, at the same time, in the opposite directions. The equilibrium between both opposite pathways is decided by the ligand attached to PGK3 [31]. This study reports nicotine as a gluconeogenesis-supporting ligand, hereby elevating the nutritional contents of the crop produce. However, PGK3 is expressed in embryo, cotyledons, and seeds, and it regulates the development of seeds and senescence [32]. To play such a prime role in the plant life cycle, it must interact with diverse metabolic factors, which have been reported in current research work, e.g., AT3G085590.2, PGM1, and AT1G79550.2. Descendants of protein PGK3 were also found to be associated with loci AT1G79550.2, AT1G12900.1, AT1G13440.1, AT3G04120.1, and AT3G26650.1. Overall, the induction of 3-phosphoglyceric acid and its direct relation with five loci (i.e., AT1G79550.2, AT1G12900.1, AT1G13440.1, AT3G04120.1, and AT3G26650.1) have been reported in the current investigation (Figure 8).

The elevated activity of nicotine-docked PGK3 affected the complete metabolism of *Vigna* through interacted chain factors. Upregulation of the complete biological process and the improved sugar metabolism, along with physiological factors (Figure 9 and Figure 10), resulted in augmented nutritional factors. In a study conducted by Huffman [33], the protein species PGK3 was enlisted among the proteins potentially regulating maize-grain nutritional contents. The current research precisely identifies PGK3 as a nutrition controlling protein in *V. unguiculata* grains, and also discovers its docking with nicotine ligand, for improved functionality. However, the improved activity of the TCA cycle (Figure 9) could be responsible for disease control, as suggested by Ahmad et al. [16].

## 5. Conclusions

It is concluded that *A. scholaris* is a natural source of a vast range of bioactive compounds and its extracts can be used for control of *M. phaseolina*. However, the present study revealed that SA was a primary antifungal defense of *Vigna* plants, and *PR2a*, acidic glucanase was the most active resistance gene. *Alstonia* derived 3-(1-methylpyrrolidin-2-yl) pyridine was responsible for improved glucanase profile and elevation of core metabolic proteins, e.g., PGK3. Moreover, twofold upregulation of cytosolic PGK3 interacted with different loci and controlled sugar metabolism and influenced the overall physiology of the plant, resulting in nutritionally rich seeds production. Further, this study can be applied in crop-cultivation programs, to get augmented yields of *V. unguiculata.*

## Supplementary Materials

The following are available online at https://www.mdpi.com/2218-273X/10/2/224/s1. Supplementary Set 1: Bioactivity-guided enrichment of the bioactive metabolite of *A. scholaris* (Figure S1). Activity pathways of phosphoglycerate kinase 3 (Figure S2).; Supplementary Data Set 2: List of total metabolites detected in *V. unguiculata*, and list of metabolites related to sugar metabolism; Supplementary Data Set 3: Gene expression and glucanase isozyme studies of *Vigna unguiculata*.; Supplementary Data Set 4: Detailed material and methods of all the experiments including plant defense assays, nutritional content studies, physiological analyses, gene expression analyses, protein profiles, isozyme experiments, physiochemical properties determination, and metabolite profiling. All reviewers and readers of the manuscript are encouraged to contact Aqeel Ahmad (aqeelahmad1@gmail.com) in case of queries.
